# Transgastric-Assisted Endoscopic Fundoplication

**DOI:** 10.1155/2013/280628

**Published:** 2013-05-13

**Authors:** Konstantinos Spaniolas, Richard I. Rothstein, Thadeus L. Trus

**Affiliations:** ^1^Section of General Surgery, Dartmouth-Hitchcock Medical Center, One Medical Center Drive, Lebanon, NH 03766, USA; ^2^Section of Gastroenterology and Hepatology, Dartmouth-Hitchcock Medical Center, One Medical Center Drive, Lebanon, NH 03766, USA

## Abstract

Gastroesophageal reflux disease (GERD) is a common entity in the United States. Surgical fundoplication can be performed safely with well-established long-term results. In selected patients with GERD, endoluminal therapy has a potential role. We report on a patient with recurrent GERD after two prior fundoplications who wished to pursue endoscopic treatment. The presence of a gastrostomy tube allowed for the performance of a transgastric-assisted endoluminal fundoplication using the EndoCinch (TM) device and standard pediatric laparoscopic instruments. Symptomatic relief of GERD with EndoCinch (TM) is common but the long-term outcomes are limited. Nevertheless, the EndoCinch (TM) device remains a method for endoscopic suturing in certain settings. In patients with gastrostomy access, the use of laparoscopic instruments may further enable the performance of advanced endoscopic therapies.

## 1. Background 

 Gastroesophageal reflux disease (GERD) represents a common problem in the United States with an estimated incidence of 28% [1]. The efficacy of surgical fundoplication is unequivocal and can be performed with minimal morbidity [2]. The development of endoluminal therapies for GERD has emerged in an effort to transorally reproduce the anatomic and physiologic results of fundoplication. Multiple devices have been developed, and a few have been introduced in clinical practice. While long-term results for most approved devices are not yet available, endoluminal therapies for GERD represent an alternative for the management of this disease. 

 The EndoCinch (TM) device (Bard Endoscopic Technologies, Murray Hill, NJ, USA) was approved for clinical use in 2000. It is placed over a standard endoscope and inserted at the level of the gastroesophageal junction through an overtube. A series of sutures are then placed in two rows, plicating the gastric wall. We report on a case where transgastric access was used to optimize application of the EndoCinch (TM) device. 

## 2. Case Report 

 We present a case of a 21-year-old male with a history of severe cerebral palsy and long-standing GERD since childhood. He had undergone two open antireflux operations, with recurrence of his symptoms of heartburn and regurgitation of tube feeds. A gastrostomy had been previously placed for nutrition intermittently. The patient's family opted to pursue endoluminal therapy. 

**Figure 1:**
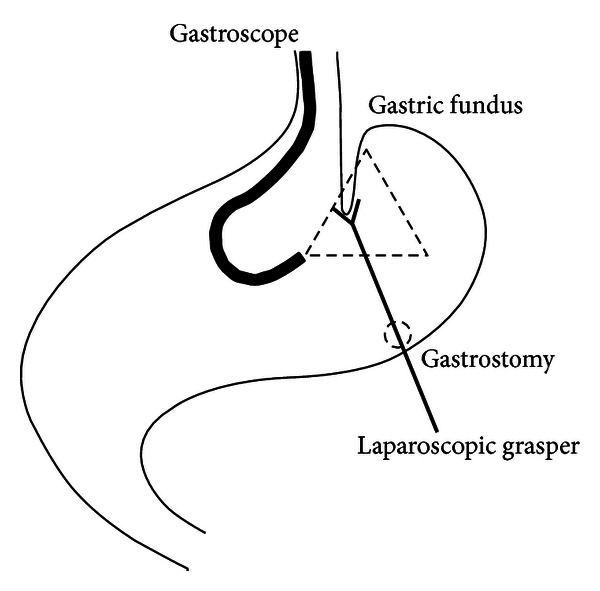
The EndoCinch (TM) device loaded on a standard gastroscope is placed transorally, while tissue is manipulated with laparoscopic graspers placed transgastrically via the gastrostomy tube.

**Figure 2:**
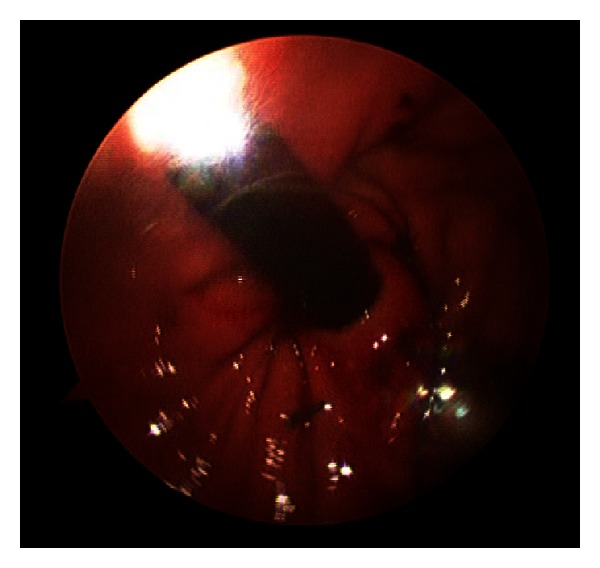
The standard gastroscope is placed through the gastroesophageal junction demonstrating intact sutures and normal valve.

 The patient underwent a partial endoluminal fundoplication performed using the EndoCinch (TM) device in the operating room. Secondary to his multiple prior antireflux procedures, a 3 mm laparoscopic grasper was placed through his gastrostomy and used for augmented transgastric retraction of the fundus during endoscopic suturing. Under direct visualization with the gastroscope, the gastric fundus was grasped and pulled away, facilitating plication (Figure 1). A 3 mm laparoscopic camera placed transgastrically via the gastrostomy tube was used for additional visualization when the grasper was not used. Only one instrument at a time was allowed via the gastrostomy. The endoscope with the attached EndoCinch (TM) system was placed in the esophagus through an overtube, and the tissue approximately 0.5 cm distal to the gastroesophageal junction was selected based on laparoscopic and endoscopic assessment. The capsule was positioned against the tissue and suction was applied to bring a fold of tissue into the chamber. The suture was deployed and the system was withdrawn, reloaded, and reapplied. A total of three plications were performed. Postprocedural inspection via the laparoscope demonstrated intact sutures with normal valve (Figure 2).

 The combined endoscopic and transgastric approach allowed for optimal and precise tissue selection for endoscopic suturing. The patient recovered uneventfully. He was observed overnight and discharged home the following day. Early followup demonstrated improvement in the patient complaints as perceived by his caregivers. However, in 3-month followup his symptoms have recurred. 

## 3. Discussion 

 Endoluminal therapies have emerged as a treatment option for GERD. The EndoCinch (TM) device was the first to be approved for the performance of endoscopic fundoplication. Initial short-term results demonstrated improved symptoms at 6 months (mean heartburn symptom scores 17 from 62.7) with improved pH monitoring and 62% of patients being virtually off acid-suppressive therapy [3]. Despite these results, 19% of patients were found to have esophagitis on repeat endoscopy. 

 Randomized control trials comparing the EndoCinch (TM) with sham have shown improvement in symptoms initially but have failed to demonstrate any long-term benefit at 12 months or improvement in pH studies. Schwartz et al. randomized 60 patients with GERD to EndoCinch (TM) fundoplication, sham, or observation [4]. They found a greater reduction in PPI use at 3 months for the treated patients, even though only 40% reported being heartburn-free. Furthermore, pH monitoring normalized in only 29% of the treated patients. At 12 months, less than a third had achieved near elimination of PPI use (defined as over 95% reduction in dose). A second randomized trial with one-year followup of 46 patients with GERD showed no difference in endoscopic findings or pH monitoring between the EndoCinch (TM) and sham groups; at 12 months, there was no difference in symptoms or PPI use [5]. 

 Despite the suboptimal long-term results with the EndoCinch (TM) device, symptomatic improvement is frequent, and the applicability of this device for endoscopic suturing remains true. Laparoscopic fundoplication remains the gold standard for interventional management of GERD, with 3.8% 30-day morbidity and 0.19% mortality [2]. However, in selected patients when an operative fundoplication is contraindicated or undesired, endoluminal therapy may represent a short-term alternative. Even though advancements in technology lead to improvements in devices, technique, and delivery systems, tissue retraction during endoluminal suturing represents a limitation. The presence of a gastrostomy allows transgastric placement of laparoscopic instruments, offering an opportunity for improved exposure and tissue manipulation.
